# Botanical Antifeedants: An Alternative Approach to Pest Control

**DOI:** 10.3390/insects16020136

**Published:** 2025-01-31

**Authors:** Roman Pavela, Kateřina Kovaříková, Matěj Novák

**Affiliations:** 1 Czech Agrifood Research Center, Drnovská 507, 161 06 Prague 6, Czech Republic; matej.novak@carc.cz; 2 Department of Plant Protection, Czech University of Life Sciences Prague, Kamýcká 129, 165 00 Prague 6, Suchdol, Czech Republic; 3 Department of Plant Biotechnology, College of Life Sciences and Biotechnology, Korea University, Seoul 02841, Republic of Korea

**Keywords:** plant extracts, antifeedant activity, essential oils, compounds, phytophagous, pests

## Abstract

Plant protection against phytophagous pests still largely relies on the application of synthetic insecticides, which can lead to environmental and health risks that are further exacerbated by the development of resistant pest populations. These are the driving forces behind the current trend of research and the development of new ecological insecticides. The mode of action does not have to rely exclusively on acute or chronic toxicity. Another promising approach is the use of plant antifeedants, which can significantly reduce the food intake of phytophagous insects. However, the information on antifeedant substances has not yet been sufficiently evaluated. The aim of this review was to find the most promising plants that provide potent extracts, essential oils (EOs), or isolated compounds with antifeedant properties. The selection was based on a comparison of effective concentrations or doses. Effective extracts were obtained from 85 plant species belonging to 35 families and the EOs came from 38 aromatic plant species from 11 families. Based on the results, *Angelica archangelica*, *Caesalpinia bonduc*, *Grindelia camporum*, *Inula auriculata*, *Lavandula luisieri*, *Mentha pulegium, Piper hispidinervum*, and *Vitis vinifera* were selected as promising plants with antifeedant potential. These plants are potent antifeedants, and at the same time provide sufficient biomass for industrial use in the development and production of botanical antifeedants.

## Simple Summary

Pests cause damage to 10–30% of agricultural production annually. To prevent this, we use plant protection products, which are mostly of synthetic origin and carry certain risks and whose use, from today’s modern point of view, is not sustainable. Plants have evolved to possess a number of mechanisms over the course of evolution that allow them to reduce damage, to some extent, from both abiotic and biotic stressors, including phytophagous insects. One of the interesting mechanisms of plant defence is the synthesis of secondary metabolites that can, among other things, inhibit food intake—a phenomenon we call antifeedance. Secondary metabolites can act on pests directly by being toxic, or by having repellent and antifeedant effects. Our review focuses on these antifeedants. By comparing the effectiveness of antifeedants from the published available research, we concluded that the most effective and promising sources of antifeedants are the following plants: *Angelica archangelica*, *Caesalpinia bonduc*, *Grindelia camporum*, *Inula auriculata*, *Lavandula luisieri, Mentha pulegium*, *Piper hispidinervum*, and *Vitis vinifera*.

## 1. Introduction

Plants are exposed to adverse conditions throughout their lives that cause them stress. Stress can be of abiotic (drought, radiation, etc.) and biotic (pests, pathogens, etc.) origin. Stress factors reduce photosynthesis and plant growth [1], seed production can also be affected [2], and the final consequence of persistent stress is the death of the entire plant. Over the course of evolution, plants have had to develop various protective mechanisms to guard their important organs from pathogens and pests. Besides morphological changes, e.g., in the form of a thicker epidermis or dense trichomes, plants have developed numerous metabolic pathways that help them synthesize protective secondary metabolites [3].

These metabolites, including terpenoids, phenols, alkaloids, cyanogenic glycosides, glucosinolates, chinons, amines, non-protein amino acids, organic acids, polyacetylenes, and peptides, may negatively affect the metabolism, neural transmission, development, and reproduction of phytophagous insects [3,4]. Green plants in particular stand out for their ability to synthesize such a wide range of secondary metabolites; even a cursory literature review documents over 100,000 compounds [5] that may affect various molecular targets at a specific time, and often synergistically [6,7]. Thus, some plant species can be described as “green chemical factories”.

However, phytophagous insects still cause annual losses estimated at 10–30%; in some cases, the yield losses can even be fatal [8]. Although the damage caused by phytophagous insects can be partially prevented by using various farming innovations (such as frequent crop rotation or the use of mixed cultures) or plant breeding (such as growing available resistant varieties), plant protection against pests is still based on the application of insecticides. Annually, approximately 2 million tonnes of pesticides are used worldwide, with insecticides accounting for just under 30% [8]. The frequent application of synthetic insecticides may cause certain environmental and health risks [9]. In addition, the development of resistant pest populations is a worldwide problem at present [10]. These and other problems have become the driving force for scientific teams to seek new active substances for insecticides, with different action mechanisms that also pose minimum or no environmental and health risks. Using secondary plant metabolites in plant protection is one of the suitable alternatives. There is a logical presumption that natural secondary metabolites will degrade rapidly in agroecosystems and, at the same time, be safe for non-target organisms, including humans [11,12].

At present, a number of botanical insecticides are on the market, usually based on plant metabolites that cause either acute (e.g., based on essential oils, extracts from *Chrysanthemum cinerariifolium*) and chronic (e.g., extracts from *Azadirachta indica* and *Pongamia pinnata*) toxicity [12].

However, secondary plant metabolites need not cause only direct insect mortality. There are numerous allomones that belong among plant metabolites. These substances do not primarily cause pest mortality, but they repel or deter insects from feeding, so that the pest will not alight on the plant, as it assesses the plant as unsuitable for feeding for itself or its offspring [5,13].

While repellent substances are currently used in the form of commercial insect repellents, particularly against noxious insects and important disease vectors such as mosquitoes and ticks [14,15], knowledge about antifeedant substances that discourage phytophagous insects from feeding is still insufficient. It is assumed that antifeedants can significantly reduce plant damage from phytophagous pests and may thus be suitable candidates for the development of “green” plant protection products [11,16,17], yet comprehensive information on promising antifeedants is still lacking.

## 2. Antifeedance Phenomenon

Antifeedants act at low concentrations and are perceived by specialized receptors [18]. Each compound that an insect tastes, i.e., each tastant, may elicit a unique spatiotemporal neural representation [13]. For simplification, tastants have been divided, similarly to human taste buds, by the category of substances they respond to, i.e., sweet, bitter, sour, and salty [19], with umami added later [20].

Sweetness is associated most importantly with sugars, but also sugar alcohols, which are important for some Lepidoptera and other insects [21]. Sweetness is an important factor, just as water content (hypoosmolarity), fatty acid content, CO_2_, and other substances are important perceptions that help insects decide whether the food is suitable for them or their offspring, and thus, whether it is appropriate to lay eggs on such plants [22].

Insect neurons, tastants, and responses to tastants are sometimes classified as appetitive, phagostimulatory or attractant versus aversive, or antifeedant deterrent or suppressant [18,23,24]. In feeding, appetitive behaviour includes biting or a proboscis extension response (PER) and ingestion. PER is the extension of retracted or folded up mouthparts in apparent preparation for contacting food and feeding, often in response to the stimulation of a body part other than the part of the mouth that will do the feeding, e.g., stimulation of the antennae, tarsi or labial palps. PER has been used to test taste in various flies [21,25], lepidopterans [26], bees [27,28], and ants [29]. Aversive behaviour includes avoidance and the inhibition of PER or biting.

Whether a compound is appetitive or aversive may differ even among closely related species [30] and within species [31]. Many aversive, primarily bitter, compounds such as alkaloids are produced by plants as repellents and antifeedants against herbivores. Subsequently, however, insect specialists have evolved and coevolved to feed on precisely these plants. They have then begun to use plant-produced repellent or antifeedant substances as a means of identifying suitability for food or the laying of eggs [32]. It is estimated, for example, that 75% of butterflies in the temperate zone and 80% in the tropic zone are food specialists (monophagous or oligophagous). Thanks to this specialization and neuroreceptor system, insects can find plants that they consider qualitatively favourable food [33]. On the other hand, searching for plants based on a specific taste and scent poses an opportunity for plant protection, assuming that we use secondary metabolites that provoke in the pest a repellent, antiovipository, or antifeedant response. If sufficiently long persistence of such effects is achieved, plant yield damage can be prevented in a targeted fashion [34,35].

According to Koul [36], there are several main groups of antifeedants. These include monoterpenes, diterpenes, coumarins, flavonoids, limonoids, and others, which have different mechanisms of action. All groups are more or less united by the fact that their effectiveness is structure related. For example, azadirachtin or salanin (limonoids) have many functional groups, and their antifeedant effect depends, among other things, on their arrangement in the molecule. Others, such as clerodane diterpenoids, occur in various isomeric forms, which essentially determine whether they will be effective or not. For coumarins (angelicin), for example, substitution of the furan ring means loss of antifeedant activity. Therefore, there is a huge variability in the effectiveness of individual substances, which are also species-specific. On the other hand, some terpenes and alkaloids have been found to be extremely effective feeding inhibitors for a number of insects, and the most potent antifeedants include azadirachtin, warburganal, strychnine, isopimpinelline, and piperenone [18].

As part of antifeedant efficacy, substances can affect the following factors:

1. Neurons associated with antifeedant receptors, which inhibit insect feeding (feeding deterring effect) [36];
2. Neurons causing the halting or slowing down of further feeding (feeding suppressing effect) [36];
3. Blocking of function of receptors stimulating herbivore feeding or binding directly to their normal feeding stimuli, such as sugars and amino acids [36]. An example of this mechanism is azadirachtin, which reduces the sensitivity of cells sensitive to sugar in herbivorous insects, thus causing the insects to incorrectly assess the nutritional suitability of treated plants [37].

“False” antifeedants are another possible type of mechanism. They are compounds that affect all the taste sensilla non-specifically (e.g., by means of immediate and general cell toxicity); therefore, they are not considered true antifeedants but rather an accompanying response to neurotoxic substances. They include some EOs and pyretroids [3,38].

Nevertheless, from the practical point of view, the mechanism of the effect is not as important, as it can be determined using electrophysical methods [17,36,39], as knowledge of doses or concentrations of extracts or their active substances that deliver an antifeedant effect that prevents a significant reduction in crop yield.

## 3. Antifeedant Efficacy Assessment Methods and Criteria

This review describes the antifeedant activity of plant extracts, plant essential oils, and isolated compounds on 30 (16 in the article and a further 14 in the Supplementary Tables) economically important insect species. All insects listed have chewing mouthparts and the selected life stage always feeds on above-ground parts of the plant (in most cases strictly or partly folivorous, exception is the granivorous larvae of *Pectinophora gossypiella*). The inhibition of food intake by antifeedants may thus be a new mechanism to prevent plant damage and thus eliminate economically significant damages.

As mentioned above, the antifeedant response can be studied using electrophysical methods, focused predominantly on measuring the neuronal response, e.g., of B2 sensilla in caterpillars [39]. From the practical point of view, it is much more convenient to use standard methods of treated and untreated leaf discs. These methods are faster, technically less demanding, and can provide more information about the importance of antifeedant efficacy in practice, and if applied in a standard way, the results of different studies can be compared.

These tests evaluate the amount of contaminated food received by insects in a given time period compared to the untreated control. This allows for the calculation of the so-called Feeding Deterrent Index (FDI), which provides information on the percentage reduction in contaminated food intake (T) compared to untreated food (C): FDI (%) = ((C − T)/(C + T)) × 100 [40].

There are two basic types of tests:

1. A choice test that utilizes the principle of insects’ ability to naturally choose food with suitable nutritional potential and not burdened with hazardous compounds. To a certain extent, insects are able to enzymatically inactivate some indigestible or poisonous substances. However, this inactivation requires energy. Additionally, some substances may inhibit their food utilization ability, and thus plants without such substances are more convenient for the insects. Nevertheless, in the absence of choice, they are able to feed on food contaminated with growth-inhibiting substances. The choice test thus answers the question of whether a substance can discourage the individuals from food intake, and the results show whether the individuals class such substances as inappropriate for feeding or whether, on the contrary, food contaminated with these substances becomes more attractive for them.
2. A non-choice test is more rigorous and provides more input for potential use in practice. If no food other than contaminated food is presented to the larvae, they are either able to feed with a time delay compared to untreated control or the treated food is unacceptable. Generally, it can be hypothesized that if the FDI is below 90%, the given substances will probably only reduce the rate of food intake, but pose no insurmountable barrier to food intake. However, an FDI value above 90% indicates that the tested compound may actually be a true antifeedant substance, as it can significantly inhibit the response of (1) olfactory receptor cells, (2) taste receptor cells, (3) oral mechanoreceptors, and/or (4) a post-ingestion response mechanism [36].

To prevent error due to uneven insect appetites, it is important to let the target insects starve for several hours (2–5 h) before commencing the experiments. It is advisable to place leaf discs in the most commonly used Petri dishes of an adequate size so that they do not touch each other, and with a spacing of at least 2–3 cm. It is best to place 3–5 larvae or adults of the target insect species in the centre of the arena. The experiment should ideally end when more than 50% (ideally up to 90%) of the control discs have been eaten. The experiment should be performed with a sufficient number of repetitions (at least 4–5) to ensure that the findings are consistent [17].

## 4. Available Literature Assessment Methods

Among other things, the present overview aimed to make a critical assessment of the results related to the antifeedant efficacy of plant extracts, Eos, and isolated substances against pests damaging plants by feeding on their leaves (defoliators). Since we intended to obtain information that would be as objective as possible and provide evidence of the primary antifeedant effects of plant secondary metabolites on a reduction in feeding, we set the following criteria for selecting appropriate publications: the use of standard choice or non-choice tests on leaf discs and known effective dose or concentration or, if the application of a single concentration had an antifeedant index above 70%, the FDI (%) was calculated using the standard formula ((C − T)/(C + T)) × 100 [40]. Publications in the WoS and Scopus databases (accessed in November 2024) were searched using the following keywords: „topic“ was set up as „antifeedant“, with exclusion of terms such as the following: anorexia, ant, aphid, aphids, aspergillus, bemisia, cellulose, coli, copper, culex, cytotoxic, diptera, fate, field, freshwater, fungal, fungi, fungus, genes, grain, hare, human, leafhopper, liver, locust, marine, metarhizium, meeting, mite, nanotechnology, oblonga, planthoppers, psyllid, quantitative, review, rhyzopertha, sea, sensory, snail, storage, stored, symposium, synthesis, termite, thrips, tribolium, and warehouse.

## 5. Promising Plant Antifeedant Substances

We identified 2803 publications in total, out of which 1280 were concerned with phyllophagous pests. Nevertheless, after meticulous study, only 286 publications matched the above criteria.

**Figure 1:**
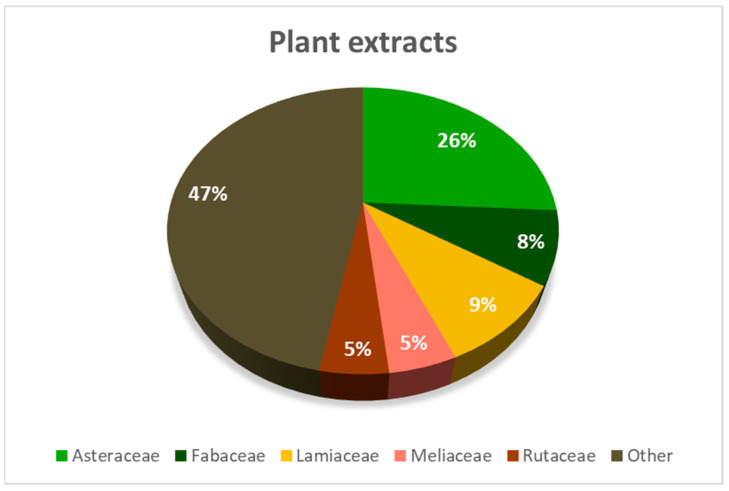
The extracts that met the efficacy criteria (minimum 70% FDI) came from 85 plant species and 35 families. However, the vast majority came from five families—*Asteraceae*, *Fabaceae*, *Lamiaceae*, *Meliaceae*, and *Rutaceae*.

Effective extracts were obtained from 85 plant species (Table S1), belonging to 35 families. Nevertheless, more than 50% of the plants belonged only to the families *Asteraceae*, *Fabaceae*, *Lamiaceae*, *Meliaceae*, and *Rutaceae* (Figure 1). Because each paper used different insect species and developmental stages, different methods of application to the surface of leaf discs, or different times of evaluation of antifeedant efficacy, etc., no single viewpoint or choice of criterion is ideal. On this basis, we have chosen EC_50_ and ED_50_ as the least misleading criteria. These values provide at least a basic idea of the antifeedant potential of the evaluated substances.

ED_50_—effective dose—is a statistically derived average dose (µg of compounds/extracts at 1 cm^2^ of the treated leaf) at which, in the case of antifeedance (as opposed to mortality), a 50% reduction in food intake compared to an untreated control is expected to be caused. EC_50_—effective concentration—is the same parameter as ED_50_, with the difference that it is not a dose per leaf area, but a concentration of the substance/extract in the solvent (organic solvent, water, etc.), usually expressed as µg ml^−1^, percent, or ppm.

If the estimated EC_50_ or ED_50_ values are taken as the main criterion, then we can highlight the chloroform extract from *Clausena anisate*, which had the lowest EC_50_ estimate (140 µg mL^−1^). It was tested in choice tests on larvae of the polyphagous pest *Helicoverpa armigera*. Furthermore, the alkaloid extracts of aerial parts of *Senecio kingii* against polyphagous larvae of *Spodoptera littoralis*, with an estimated ED_50_ of 0.09 μg cm^−2^, and *Vitis vinifera* shoots extracts against oligophagous adults of *Leptinotarsa decemlineata*, with an estimated ED_50_ of 0.08 μg cm^−2^ (Table 1), were tested.

The majority of substances identified in the extracts were coumarins, flavonoids, terpenoids, phenols, and quinones. Other promising extracts that showed ED_50_ ˂ 1 µg cm^−2^ were obtained from *Vitis vinifera*, *Senecio kingii*, *Grindelia camporum*, *Inula auriculata*, *Angelica archangelica*, *Echium wildpretii* subsp. *wildpretii*, and *Senecio fistulosus*. Their antifeedant efficacy was tested both on the oligophagous *Leptinotarsa decemlineata* and the polyphagous *Spodoptera littoralis* (Table 1). Very good efficacy has also been demonstrated by extracts obtained from *Azadirachta indica*, *Melia volkensii*, *Pyrethrum corymbosum*, *Teucrium hircanicum*, *Xeranthemum cylindraceum*, and *Persea indica* (ED_50_ was determined at 1–10 µg cm^−2^). Although complete analyses of all these most effective extracts were not conducted, the following majority substances were identified in some of them: eremophilane-type sesquiterpenes of the furanoeremophilane, as well as furanocoumarins (bergapten, imperatorin, and phellopterin), limonoids (e.g., azadirachtin), and other terpenoids (Table 1).

**Figure 2:**
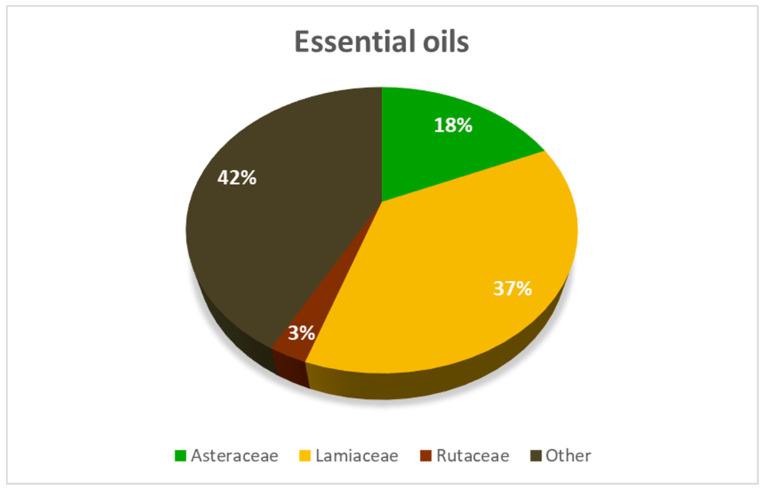
Essential oils with an FDI of at least 70% came from a total of 38 plant species and 11 families. The majority of these plants belonged to three families—*Asteraceae*, *Lamiaceae*, and *Rutaceae*.

A total of 38 aromatic plant species belonging to 11 families yielded EOs with promising antifeedant effects (Table S2). More than 50% of these plant species, however, belonged to only three families: *Asteraceae*, *Lamiaceae*, and *Rutaceae* (Figure 2).

The most effective EOs had ED_50_ ˂ 10 µg cm^−2^. Those EOs were obtained from *Acorus calamus* (majority compound—asarone), which was tested against *Peridroma saucia*. Other promising EOs were tested against larvae of *Spodoptera littoralis* or *S. littura*. Those EOs were obtained from *Angelica archangelica* (majority compounds—β-phellandrene, sabinene, α-pinene, and α-phellandrene), *Lavandula luisieri* (majority compounds—3-Oxo-cadinol, 2,3,4,4-Tetramethyl-5-methylidenecyclopent-2-en-1-one, and Hydroxymethyl-2,3,4,4-tetramethylcyclopent-2-en-1-one), *Mentha pulegium* (majority compounds—pulegone, 1,3,4-trimethyl-3-cyclohexene-1-carboxaldehyde, and piperitenone), *Artemisia nakaii* (majority compounds—Feropodin, (+)-camphor, 1,8-cineole, and rishitin), *Piper hispidinervum* (majority compounds—safrole and terpinolene) and *Piper sanctifelicis*, which was tested against *Leptinotarsa decemlineata* (majority compounds—δ-3-carene, limonene, p-cymene, β-pinene, and nerolidol) (Table 2).

More than 100 isolated compounds and their derivatives were tested for antifeedant efficacy against several species of important phytophagous pests (Table S3). Pure compounds often showed stronger efficacy compared to extracts or EOs. Several isolated plant substances (floral diterpenoids, homopterocarpin, anhydrocinnzeylanine, cinnzeylanine, silphinene, pulegone, thujone, ursane type triterpenoid, aconitine, and xanthotoxin) even had an estimated ED_50_ ˂ 1 µg cm^−2^. Some other substances had ED_50_ values of 1–10 µg cm^−2^ (compound 1—latex diterpenoids, piperine, ursane type triterpenoid, rocaglamide, volkensin, grayanane diterpenoids, uvedalin, toosedanin, 6-methylflavone, pinocembrin, polygodial, ligudicin A, piperitenone epoxide, α-pinene, dehydrofukinone, germacrone, neo-clerodane diterpenoids, β-ocimene, derivatives of eugenol, and thymol) (Table 3).

The tables list the 30 species of insects tested. The majority of these are representatives of the order Lepidoptera (22 species), including 5 oligophagous species (*Cnaphalocrocis medinalis*, *Pectinophora gossypiella*, *Pieris brassicae*, *P. rapae*, and *Plutella xylostella*) and 17 polyphagous species (*Achaea janata*, *Crocidolomia pavonana*, *Helicoverpa armigera*, *Hyphantria cunea*, *Lymantria dispar*, *Mamestra brassicae*, *M.* *configurata*, *Ostrinia furnacalis*, *O. nubilalis*, *Peridroma saucia*, *Spilosoma obliqua*, *Spodoptera exempta, S. frugiperda*, *S. littoralis*, *S. litura S. exigua*, and *Trichopulsia ni*).

Furthermore, seven representatives of the order Coleoptera were represented, of which the larvae of three species are oligophagous (*Diabrotica barberi*, *Epilachna paenulata*, and *Leptinotarsa decemlineata*) and four are polyphagous (*Diabrotica undecimpunctata*, *D. virgifera*, *Epicauta atomaria*, and *Epilachna varivestis*).

The order Hymenoptera was represented by one oligophagous species (*Caliroa cerasi*) (listed only in the Supplementary Tables).

The best effect on oligophagous Lepidoptera larvae was observed with the extract of *Panax ginseng* (EC_50_ for *P. xylostella* estimated at 2740 μg mL^−1^) and the essential oil from *Citrus aurantifolia* (ED_50_ for *P. xylostella* estimated at 68.93 μg cm^−2^). The most promising compounds were isolated from *Pieris japonica* (flower diterpenoids; ED_50_ for *P. brassicae* estimated at 0.028 μg cm^−2^), *Croton jatrophoides* (dumnin and dumsenin; EC_50_ for *P. gossypiella* estimated at ≤2 μg mL^−1^), *Rhododendron molie* extract (rhodojaponin III; EC_50_ for *P. rapae* estimated at 1.16 μg mL^−1^), and, from an unspecified plant, the derivatives of eugenol and thymol (EC_50_ for *P. xylostella* estimated at 3.3 μg mL^−1^).

The best effect on polyphagous lepidopteran larvae was observed with extracts of *Angelica archangelica* (ED_50_ for *S. littoralis* estimated at 0.64 μg cm^−2^), *Senecio fistulosus* (ED_50_ for *S. littoralis* estimated at 0.64 μg cm^−2^), and *Clausena anisata* (EC_50_ for *H. armigera* estimated at 140 μg mL^−1^). A significant antifeedant effect for these pests was observed with the essential oil from *Acorus calamus* (ED_50_ for *P. saucia* estimated at 2.5 μg cm^−2^), *Artemisia nakaii* (ED_50_ for *S. litura* estimated at 3.76 μg cm^−2^), and *Mentha pulegium* (ED_50_ for *S. littoralis* estimated at 1.3 μg cm^−2^). The most promising compounds were isolated from *Eupatorium adenophorum* (sesquiterpenoids—compound 2 and 3; ED_50_ for *H. armigera* estimated at 2.5 and 3.0 μg cm^−2^), *Senecio adenotrichius* (compound 1—dehydrofukinone; ED_50_ for *S. litoralis* estimated at 1.6 μg cm^−2^), and *Euphorbia paralias* (ursane type triterpenoid—compound 21 (uvaol); ED_50_ for *S. litoralis* estimated at 3.3 μg cm^−2^).

The highest antifeedant effect on the oligophagous coleopteran larvae was shown by the extract of *Angelica archangelica* (ED_50_ for *L. decemlineata* larvae estimated at 0.6 μg cm^−2^) and *Echium wildpretii* (ED_50_ for *L. decemlineata* (unspecified stadium) estimated at 0.4 μg cm^−2^). The most promising essential oil for these pests is *Piper hispidinervum* EO (ED_50_ for *L. decemlineata* (unspecified stadium) estimated at 0.4 μg cm^−2^) and the most promising isolated compounds are from *Euphorbia paralias* (ursane type triterpenoid—compound 21 (uvaol); ED_50_ for *L. decemlineata* adults estimated at 0.42 μg cm^−2^), *Persea indica* (cinnzeylanone; ED_50_ for *L. decemlineata* adults estimated at 0.08 μg cm^−2^), and from the unspecified plant seskviterpene silphinene (ED_50_ for *L. decemlineata* adults estimated at 0.15 μg cm^−2^).

The best effect on polyphagous coleopteran larvae was observed with extracts of *Melia volkensii* (ED_50_ for *E. varivestis* adults estimated at 2.3 μg cm^−2^) and the alkaloid aconitine from an unspecified plant (ED_50_ for *D. virgifera* adults estimated at 0.27 μg cm^−2^).

The results of our study show that a number of plants contain secondary metabolites that demonstrate promising antifeedant efficacy against many important pests (e.g., Leptinotarsa *decemlineata*, *Spodoptera littoralis*, *S. litura*, *Trichopulsia ni*, *Helicoverpa armigera*, *Plutella xylostella*, *Pieris rapae*, and *Ostrinia nubilalis,* etc.). In terms of the practical implementation of the research results in terms of new products with antifeedant activity, those extracts that show antifeedant efficacy in the lowest possible concentrations or doses can be regarded as especially promising. Our paper has identified numerous extracts with estimates of ED_50_ < 10 µg cm^−2^, which in the standard application of 10 µL application liquid per cm^2^ [44,45], corresponds to a concentration below 0.1%; it can therefore be assumed that a concentration 2–3 times higher will, in the majority of cases, cause maximum inhibition of feeding in the pests (Tables S1–S3). Another prerequisite for the practical implementation of antifeedants is the safety of the applied substances for both the environment and non-target organisms, including humans. This safety can be primarily estimated, e.g., based on ethnobotanical experience of using selected plants in the food industry or on the known safety of the contained substances. In spite of that, however, it will be necessary to perform appropriate biological testing, particularly for the effects of the selected extracts on non-target organisms.

Another prerequisite for the selection of extracts suitable for the development of insect antifeedants is the availability and easy cultivation of plants that can provide sufficient amounts of biomass suitable for the extraction of active substances [101]. In our paper, we selected the following highly effective plants: *Caesalpinia bonduc*, *Vitis vinifera*, *Senecio kingii*, *Grindelia camporum*, *Inula auriculata*, *Angelica archangelica*, *Echium wildpretii* subsp. *wildpretii*, and *Senecio fistulosus*. Among them, *C. bonduc*, *V. vinifera*, *G. camporum*, *I. auriculata*, and *A. archangelica* can be chosen as highly promising plants because they are frequently grown as medicinal or cultural crops and can provide the biomass suitable for the extraction of active substances. Likewise, EOs from *Angelica archangelica*, *Lavandula luisieri*, *Mentha pulegium*, and *Piper hispidinervum* can be suitable for the development of new products with antifeedant activity. Nevertheless, from a practical point of view, the stability of active substances in the environment is no less important, enabling persistent effects [34,35]. From this perspective, plant extracts appear more promising than EOs, which show considerable instability [102]. Nevertheless, in this case, the solution might consist of protecting the molecules and releasing them gradually, using the appropriate encapsulation methods [103]. However, tests that demonstrate the persistence of the antifeedant effect over time are lacking.

## 6. Future Research Challenges

Currently, studies on antifeedance are among the most frequently used auxiliary biological tests in the study of the chronic toxicity of plant metabolites. The chronic mortality of insects can occur over time due to low food intake and subsequent low conversion to weight gain [104]. Therefore, the study of antifeedance is very important. As can be seen from the results of this work, there are many plants that contain substances with high antifeedant potential. These substances can be useful in plant protection. The mortality of such pests can be a secondary effect.

Although we managed to select promising plants based on the comparison of effective concentrations or doses, we must admit that comparing results across authors is fraught with a certain amount of error. This error results mainly from the heterogeneity of the methods used by different authors. At the same time, it is important to emphasize that these tests are often short-term and do not indicate the persistence of the effect. Systematic research dealing with the phenomenon of antifeedance is still lacking.

These are the potential challenges for future research, which refer to the aim of implementing these results into agronomical practice:

1. Primary tests. The aim of primary tests is to select plants that contain substances with antifeedant potential. Within these tests, it is therefore necessary to unify the methods and use the generally accepted method of no-choice tests on leaf discs, because it most closely simulates the likely efficacy in agricultural practice [17,36,39]. For calculating the FDI and estimating effective concentrations or doses, it is important to use the above formula [40] to ensure better comparability of results. Only standardized methods and procedures that are statistically valid can provide results that can be compared between different laboratories.
2. Expand knowledge about the efficacy of extracts. Another set of research goals is undoubtedly follow-up tests to the “Primary tests”, which should aim to find out other important information about the effectiveness of selected antifeedants. a) For extracts and EOs, it is important to find out which substances or their combinations cause the antifeedant effect, which is important information from the point of view of standardization of extracts as active substances of potential plant protection products. b) Study of the effect of extracts and active substances on non-target organisms, which is important information for estimating their environmental safety. c) Study of the persistence of the effect and synergetic relationships of the majority substances with regard to a possible increase in effectiveness or extension of the persistence period.
3. Implementation of results, i.e., transfer of results from laboratory experiments to agricultural practice. It is important that scientific knowledge is put into practice in the form of plant protection preparations. a) It is therefore important to systematically investigate formulation methods that will extend the period of effectiveness (e.g., encapsulation methods, etc.). b) It is important to verify the effects under conditions simulating the real ones, that is, the translation of knowledge into practice by applying formulated products in container and field trials and comparing their efficacy with other insecticides, especially on the yield characteristics of treated crops.

Field experiments are few in number compared to the abundance of laboratory studies. Laboratory studies are relatively straightforward and tend to produce significant results that can be easily published, while field studies are more difficult to conduct, much more variable, and often show poor efficacy of the test substance compared to established plant protection products.

## 7. Conclusions

Based on the comparison of effective concentrations or doses of extracts/essences and substances, we selected some plants that contain highly promising secondary metabolites. Among them, there are extracts from *Caesalpinia bonduc*, *Vitis vinifera*, *Grindelia camporum*, *Inula auriculata*, and *Angelica archangelica*, and EOs used as antifeedants can be obtained from the aromatic plants *Angelica archangelica*, *Lavandula luisieri*, *Mentha pulegium*, and *Piper hispidinervum*. Thus, these plant species can be the subject of further research and a potential source of active ingredients for new plant protection products with antifeedant activity.

## Figures and Tables

**Table 1:** Plant extracts showing significant antifeedant effect; selection based on effective concentrations or doses. Insect: L = larvae (instar) and A = adults. The concentration is given in µg mL^−1^ for liquids, or in µg cm^−2^ for the dose of active ingredient calculated on a given leaf surface area. The resulting value also corresponds to this: EC_50_ for liquids or ED_50_ for solids.

Family/Plant	Type	Majority Compounds with Antifeedant Effect	Insect (Instar)	Test	EC_50_ (μg mL^−1^)	ED_50_ (μg cm^−2^)	References
Anacardiaceae							
*Anacardium occidentale* L.	Cashew nutshell liquid	Phenolic compounds	*Spodoptera frugiperda*—L4	no choice	3400		[41]
Annonaceae							
*Annona squamosa* L.	Crude seed methanol extracts	Sesquiterpenes, monoterpenes	*Trichoplusia ni*—L3	choice	2300		[42]
*Polyalthia longifolia* (Sonn.) Thwaites	Methanol extract	Terpenes	*Spodoptera litura*—L3	no choice	1080		[43]
Apiaceae							
*Angelica archangelica* L.	Methanol extracts	Unspecified	*Leptinotarsa decemlineata*—L4	no choice		0.6	[44]
*Angelica archangelica* L.	Seeds benzene extract	Bergapten, imperatorin, phellopterin	*Spodoptera littoralis*—L3	no choice		0.31	[45]
*Angelica archangelica* L.	Seeds acetone extract	Bergapten, imperatorin, phellopterin	*Spodoptera littoralis*—L3	no choice		0.65	[45]
*Angelica archangelica* L.	Seeds methanol extract	Bergapten, imperatorin, phellopterin	*Spodoptera littoralis*—L3	no choice		0.54	[45]
Apocynaceae							
*Tylophora indica* (Burm. f.) Merr.	Ethanolic extract	Unspecified	*Spodoptera litura*—L4		8300		[46]
Araliaceae							
*Panax ginseng* C. A. Meyer	Stems and leaves extract—ginsenosides	Ginsenosides	*Plutella xylostella*—L2	choice	2740		[47]
Asteraceae							
*Grindelia camporum* Hook. & Arn.	Methanol extracts	Unspecified	*Leptinotarsa decemlineata*—L4	no choice		0.2	[44]
*Inula auriculata* Boiss. & Balansa	Methanol extracts	Unspecified	*Leptinotarsa decemlineata*—L4	no choice		0.2	[44]
*Pyrethrum corymbosum* (L.) Scop.	Methanol extracts	Unspecified	*Leptinotarsa decemlineata*—L4	no choice		3	[44]
*Senecio fistulosus* Poepp. ex DC	Furanoeremophilane	Eremophilane-typesesquiterpenes of the furanoeremophilane and eremophilanolidesesquiterpenes	*Spodoptera littoralis*—L6			0.64	[48]
*Senecio kingii* Hook.f.	Aerial parts alkaloidal extracts	Eremophilanolidess, shikimic acid derivatives, flavonoids	*Spodoptera littoralis*—L6	choice		0.09	[49]
*Xeranthemum cylindraceum* Sibth. & Sm.	Methanol extracts	Unspecified	*Leptinotarsa decemlineata*—L4	no choice		8	[44]
Boraginaceae							
*Echium wildpretii* H. Pearson ex Hook. f. subsp. *wildpretii*	Fraction 2 from ethanol extract: hexane/ethyl acetate, 90: 10 *v*/*v*); steroidal fraction—compound 3	Fatty acid esters, phytosterols	*Leptinotarsa decemlineata* (unspecified stadium)	choice		0.4	[50]
Fabaceae							
*Caesalpinia bonduc* (L.) Roxb.	Chloroform extract—fraction 3	Coumarins, flavonoids, terpenoids, phenols, quinones	*Helicoverpa armigera*—L3	no choice	357.13		[51]
*Millettia pachycarpa* (Benth.)	Fresh leaves methanol extract -> dichlormethane -> fraction 2	Triterpenoid (lupeol)	*Spodoptera litura*—L3	no choice	227.13		[52]
Lamiaceae							
*Teucrium hircanicum* L.	Methanol extracts	Unspecified	*Leptinotarsa decemlineata*—L4	no choice		6	[44]
Lauraceae							
*Persea indica* (L.) Spreng.	Stem extract	Ryanoids	*Spodoptera littoralis*—L6	no choice		8.5	[53]
Meliaceae							
*Melia volkensii* Gürke	Refined seed extract	Terpenoids	*Epilachna varivestis*—A	choice		2.3	[54]
Rutaceae							
*Clausena anisata* (Willd.) Hook.f. ex Benth.	Chloroform root extracts	Osthol (coumarin derivate)	*Helicoverpa armigera*—L5	choice	140		[55]
*Clausena anisata* (Willd.) Hook.f. ex Benth.	Petroleum ether root extracts	Osthol (coumarin derivate)	*Helicoverpa armigera*—L5	choice	160		[55]
Solanaceae							
*Solanum xanthocarpum* Schrad. & Wendl.	Chloroform extract, fraction 4	Terpenoids, flavonoid, quinone	*Helicoverpa armigera*—L3	no choice	378.3		[56]
Vitaceae							
*Vitis vinifera* L.	Vine-shoot wastes: Conventional Solid–Liquid Extraction 60 min	Flavanols	*Leptinotarsa decemlineata*—A	choice		0.08	[57]

**Table 2:** Plant essential oils showing significant antifeedant effect; selection based on effective concentrations or doses. Insect: L = larvae (instar) and A = adults. The concentration is given in µg mL^−1^ for liquids, or in µg cm^−2^ for the dose of active ingredient calculated on a given leaf surface area. The resulting value also corresponds to this: EC_50_ for liquids or ED_50_ for solids.

Family/Plant	Parts	Majority Compounds with Antifeedant Effect	Insect (Instar)	Test	EC_50_ (μg mL^−1^)	ED_50_ (μg cm^−2^)	References
Acoraceae							
*Acorus calamus* L.	Rhizomes—cis-asarone	Cis-asarone, trans-asarone	*Peridroma saucia*—L4	choice		2.5	[58]
Apiaceae							
*Angelica archangelica* L.	Seeds	β-Phellandrene, sabinene, α-pinene, α-phellandrene	*Spodoptera littoralis*—L3	no choice		7.12	[45]
Asteraceae							
*Artemisia nakaii* Pamp.	Aerial parts	Feropodin, (+)-camphor, 1,8-cineole, rishitin	*Spodoptera litura*—L3	choice		3.76 ± 0.73	[59]
Lamiaceae							
*Lavandula luisieri* (Rozeira)—cultivated pop.	Flowering parts	3-Oxo-cadinol, 2,3,4,4-Tetramethyl-5-methylidenecyclopent-2-en-1-one, Hydroxymethyl-2,3,4,4-tetramethylcyclopent-2-en-1-one	*Spodoptera littoralis*—L6			10.23	[60]
*Mentha pulegium* Mill.	Leaves and flowers	Pulegone, 1,3,4-trimethyl-3-cyclohexene-1-carboxaldehyde, piperitenone	*Spodoptera littoralis*—L6	choice		1.3 (0.4, 4.1)	[61]
Piperaceae							
*Piper hispidinervum* C.DC.	Fresh leaves and twigs	Safrole, terpinolene	*Leptinotarsa decemlineata*	choice		0.4	[62]
*Piper hispidinervum* C.DC.	Fresh leaves and twigs	Safrole	*Spodoptera littoralis*—L	choice		3.1	[62]
*Piper sanctifelicis* Trel.	Leaves	δ-3-carene, limonene, p-cymene, β-pinene, nerolidol	*Spodoptera littoralis*—L6	choice		4.5 (4.3–4.7)	[63]
Rutaceae							
*Citrus aurantifolia* (L.) Swingle	Commercial EOs and limonene	γ-Muurolene, o-cymene, bornyl acetate, α-bisabolol	*Plutella xylostella*—L3—deltamethrin susceptible strain	choice		68.93	[64]

**Table 3:** Isolated compounds showing significant antifeedant effect; selection based on effective concentrations or doses. Insect: L = larvae (instar) and A = adults. The concentration is given in µg mL^−1^ for liquids, or in µg cm^−2^ for the dose of active ingredient calculated on a given leaf surface area. The resulting value also corresponds to this: EC_50_ for liquids or ED_50_ for solids.

Plant	Type	Insect (Instar)	Test	EC_50_ (μg mL^−1^)	ED_50_ (μg cm^−2^)	References
Asteraceae						
*Carpesium abrotanoides* L.	Air-dried fruits—compound 1	*Plutella xylostella*—L3	choice	19.8		[65]
*Eupatorium adenophorum* Spreng	Sesquiterpenoids, compound 2 and 3	*Helicoverpa armigera*—L2	choice		2.5 and 3.0	[66]
*Flourensia oolepis* S.F. Blake	Aerial parts—flavonoid pinocembrin	*Epilachna paenulata*—L3	choice		10	[67]
*Pericallis* spp.	3-ethoxy-hydroxy-tremetone; (-)-eupachinin A	*Spodoptera litoralis*—L6	choice	130		[68]
*Senecio adenotrichius* DC.	Compound 1—dehydrofukinone	*Spodoptera litoralis*—L6	unspecified		1.6	[69]
*Senecio palmensis* C. Sm.	11*β*, 5α-dihydroxysilphinen-3-one	*Leptinotarsa decemlineata*—L4	no choice		11.3	[70]
*Smallanthus sonchifolius* (Poepp. & Endl.) H. Rob	Uvedalin	*Spodoptera litura*—L3	choice		8	[71]
Colchicaceae						
*Gloriosa superba* L.	Chloroform tuber extract—GST4	*Spodoptera litura*—L3	no choice	26		[72]
Cucurbitaceae						
*Citrullus colocynthis* (L.) Schrad.	Cucurbitacin E—fruits (fraction III)	*Spodoptera litura*—L5	choice	24.1		[73]
Cupressaceae						
*Juniperus sabina* L.	Petroleum ether extract—deoxypodophyllotoxin (1)	*Pieris rapae*—L5		60 (48 h)		[74]
Ericaceae						
*Pieris formosa* (Wallich) D. Don	Grayanane diterpenoids—10	*Spodoptera exigua*	choice modified		6.58	[75]
*Pieris japonica* (Thunb.) D. Don ex G. Don	Neopierisoid B—isolated from flowers	*Pieris brassicae*—L3	choice		5.33	[76]
*Pieris japonica* (Thunb.) D. Don ex G. Don	Flower diterpenoids—C10	*Pieris brassicae*—L3	choice		0.03	[77]
*Rhododendron molle* (Blume) G. Don	Rhodojaponin III—grayanoid diterpene from flowers	*Pieris rapae*—L3	no choice	1.16		[78]
*Rhododendron molle* (Blume) G. Don	Rhodojaponin III—grayanoid diterpene from flowers	*Pieris rapae*—L5	no choice	15.85		[78]
Euphorbiaceae						
*Croton jatrophoides* Pax.	Limonoids dumnin, dumsenin	*Pectinophora gossypiella*—L2 *Spodoptera frugiperda*—L2	choice	≤2		[79]
*Croton jatrophoides* Pax.	Limonoids from methanol extract—Musidunin	*Pectinophora gossypiella*—L2	choice	3		[80]
*Croton jatrophoides* Pax.	Limonoids from methanol extract—Musiduol	*Pectinophora gossypiella*—L2	choice	4		[80]
*Croton jatrophoides* Pax.	Limonoids from methanol extract—Musiduol	*Spodoptera frugiperda*—L2	choice	2		[80]
*Euphorbia paralias* L.	Ursane type triterpenoid—compound 21 (uvaol)	*Leptinotarsa decemlineata*—A	choice		0.2	[81]
*Euphorbia paralias* L.	Ursane type triterpenoid—compound 21 (uvaol)	*Spodoptera litoralis*—L6	choice		3.3	[81]
Fabaceae						
*Caesalpinia bonduc* (L.) Roxb.	Chloroform extract	*Helicoverpa armigera*—L3	no choice	1.08		[56]
*Pterocarpus macrocarpus* Kurz	Homopterocarpin	*Spodoptera litura*—L3	choice		0.04	[82]
Lamiaceae						
*Clerodendrum infortunatum* L.	Clerodane diterpenoids—compound 1	*Helicoverpa armigera*—L3	choice	6		[83]
Lauraceae						
*Persea indica* (L.) Spreng.	Anhydrocinnzeylanine	*Spodoptera littoralis*—L5	choice		0.09	[84]
*Persea indica* (L.) Spreng.	Anhydrocinnzeylanine	*Leptinotarsa decemlineata*—A	choice		0.94	[84]
*Persea indica* (L.) Spreng.	Cinnzeylanine	*Spodoptera litoralis*—L5	choice		0.004	[85]
*Persea indica* (L.) Spreng.		*Leptinotarsa decemlineata*—A	choice		0.08	[85]
	Cinnzeylanone					
Meliaceae						
*Aglaia odorata* Lour.	Rocaglamide from dried twigs	*Peridroma saucia*—L4	choice		3.45	[86]
*Azadirachta indica* A. Juss.	Azadirachtin	*Ostrinia nubilalis*—L1 and L3	no choice	3.5 and 24		[87]
*Melia volkensii* Gürke	Volkensin	*Spodoptera frugiperda*—L3	choice		3.5	[88]
*Melia volkensii* Gürke	Xanthotoxin 99%	*Trichopulsia ni*—L3	choice		0.9	[54]
*Melia toosendan* Siebold & Zucc.	Toosedanin—limonoid isolated from bark	*Peridroma saucia*—L4	choice		8.04	[89]
Piperaceae						
*Piper ribesioides* Wall.	Piperine	*Spodoptera litura*—L3	choice		3.1	[90]
Plantaginaceae						
*Linaria saxatilis* (L.) Chaz.	Neo-clerodane diterpenoids, compound 2	*Leptinotarsa decemlineata*—A	choice/no choice		10.5/8.5	[91]
*Linaria saxatilis* (L.) Chaz.	Neo-clerodane diterpenoids, compound 6	*Leptinotarsa decemlineata*—A	choice/no choice		12.8/7.7	[91]
*Linaria saxatilis* (L.) Chaz.	Neo-clerodane diterpenoids, compound 8	*Leptinotarsa decemlineata*—A	choice		6.4	[91]
Rutaceae						
*Citrus aurantiifolia* (Christm.) Swingle	Limonene	*Plutella xylostella*—L3—deltamethrin susceptible strain	choice	4.44		[92]
*Citrus aurantiifolia* (Christm.) Swingle	Limonene	*Plutella xylostella*—L3—deltamethrin resistant strain	choice	17.83		[92]
Simaroubaceae						
*Eurycoma longifolia* Jack	Eurycomanone	*Plutella xylostella*—L3	choice	14.2		[93]
Winteraceae						
*Drimys winteri* J.R. Forster et G. Forster	Polygodial	*Spodoptera frugiperda*—L3	choice		5.59	[94]
Unspecified						
	α-Pinene	*Spodoptera litura*—L3	no choice		1.13 uL cm^−2^	[95]
	Aconitine	*Diabrotica virgifera*—A	choice		0.27	[96]
	Dehydrofukinone (SO)	*Spodoptera littoralis*—L6	choice		1.68	[69]
	Derivatives of eugenol and thymol (6)	*Plutella xylostella*—L3	choice	4.29		[97]
	Derivatives of eugenol and thymol (8)	*Plutella xylostella*—L3	choice	3.3		[97]
	Derivatives of eugenol and thymol (10)	*Plutella xylostella*—L3	choice	6.52		[97]
	Derivatives of eugenol and thymol (thymol)	*Plutella xylostella*—L3	choice	6.38		[97]
	Germacrone (SO)	*Spodoptera littoralis*—L6	choice		1.9 (0.1–3.6)	[98]
	Piperitenone epoxide	*Spodoptera littoralis*—L6	choice		0.18 (0.01, 3.0)	[99]
	Pulegone	*Spodoptera littoralis*—L6	choice		0.2	[61]
	Pulegone	*Spodoptera littoralis*—L6	choice		0.25	[99]
	Silphinene	*Leptinotarsa decemlineata*—A	choice		0.15	[96]
	Thujone	*Spodoptera littoralis*—L6	choice		0.2	[61]
	11α-Epoxy-eremophil-9-en-8-one (ligudicin A)1	*Spodoptera littoralis*—L6	choice		0.08 (0.04–0.18)	[69]
	(E)-β-Ocimene	*Spodoptera littoralis*—L6	choice		10.6 (7.1, 15.9)	[100]

## Data Availability

There are no additional data, as all data are presented in the paper or Supplementary Materials.

## Supplementary Materials

The following supporting information can be downloaded at: https://www.mdpi.com/article/10.3390/insects16020136/s1. Table S1: Antifeedant efficacy of plant extracts (full version with FDI > 70%); Insect: L = larvae (instar) and A = adults; The concentration is given in μg mL^−1^ for liquids, or in μg cm^−2^ for the dose of active ingredient calculated on a given leaf surface area, the resulting value also corresponds to this: EC_50_ for liquids or ED_50_ for solids. Table S2: Antifeedant efficacy of plant essential oils (full version with FDI > 70%); Insect: L = larvae (instar) and A = adults; The concentration is given in μg mL^−1^ for liquids, or in μg cm^−2^ for the dose of active ingredient calculated on a given leaf surface area, the resulting value also corresponds to this: EC_50_ for liquids or ED_50_ for solids. Table S3: Antifeedant efficacy of isolated compounds (full version with FDI > 70%); Insect: L = larvae (instar) and A = adults; The concentration is given in μg mL^−1^ for liquids, or in μg cm^−2^ for the dose of active ingredient calculated on a given leaf surface area, the resulting value also corresponds to this: EC_50_ for liquids or ED_50_ for solids [105,106,107,108,109,110,111,112,113,114,115,116,117,118,119,120,121,122,123,124,125,126,127,128,129,130,131,132,133,134,135,136,137,138,139,140,141,142,143,144,145,146,147,148,149,150,151,152,153,154,155,156,157,158,159,160,161,162,163,164,165,166,167,168,169,170,171,172,173,174,175,176,177,178,179,180,181,182,183,184,185,186,187,188,189,190,191,192,193].
