# Supplementary material for: Botanical Antifeedants: An Alternative Approach to Pest Control
Source: Insects. 2025 Jan 31;16(2):136. doi: 10.3390/insects16020136 (PMC11855740; doi:10.3390/insects16020136)
Supplement: Supplementary file 1 [file insects-16-00136-s001.zip › insects-3393974-supplementary.pdf]

Table S1. Antifeedant efficacy of plant extracts (full version with FDI > 70%); Insect: L = larvae (instar) and A = adults; The concentration is given in μg mL^{-1} for liquids, or in μg cm^{-2} for the dose of active ingredient calculated on a given leaf surface area, the resulting value also correspods to this: EC_{50} for liquids, or ED_{50} for solids.

**Family/Plant**	**Type**	**Majority compounds with antifeedant effect**	**Insect (instar)**	**Test**	**Concentration (μg mL^{-1})**	**Dose (μg cm^{-2})**	**FDI (%) ± SE**	**EC_{50} (μg mL^{-1})**	**ED_{50} (μg cm^{-2})**	**References**
Acanthaceae										
Adhatoda vasica Nees	Leaf extract	Flavonoids, terpenoids, hydroxyketones, alkaloids	*Spodoptera littoralis* - L6	choice	2000		90.4 ± 11.6			105
Anacardiaceae										
Anacardium occidentale L.	Cashew nutshell liquid	Phenolic compounds	*Spodoptera frugiperda* - L4	no choice	5000–70000		45.02–98.56	3400		41
Annonaceae										
Annona squamosa L.	Crude seed methanol extracts	Sesquiterpenes, monoterpenes	*Trichoplusia ni* - L3	choice	4000		60	2300		42
Polyalthia longifolia (Sonn.) Thwaites	Methanol extract	Terpenes	*Spodoptera litura* - L3	no choice				1080		43
Apiaceae										
Angelica archangelica L.	Plant extracts	Angelicin	*Spodoptera littoralis* - L4	no choice		500	98.9		44	44
Angelica archangelica L.	Methanol extracts	Unspecified	*Leptinotarsa decemlineata* - L4	no choice		500	100		0.6	44
Angelica archangelica L.	Seeds benzene extract	Bergapten, imperatorin, phellopterin	*Spodoptera littoralis* - L3	no choice					0.31	45
Angelica archangelica L.	Seeds acetone extract	Bergapten, imperatorin, phellopterin	*Spodoptera littoralis* - L3	no choice					0.65	45
Angelica archangelica L.	Seeds methanol extract	Bergapten, imperatorin, phellopterin	*Spodoptera littoralis* - L3	no choice					0.54	45
Imperatoria ostruthium L.	Plant extracts	Unspecified	*Spodoptera littoralis* - L4	no choice		500	99.8		34	44

Imperatoria ostruthium L.	Plant extracts		*Leptinotarsa decemlineata* - L4	no choice		500	100.0		27	44
Apocynaceae										
Pergularia daemia (Forssk.) Chiov.	Ethyl acetate extract - leaves	Quinones	*Helicoverpa armigera* - L4	no choice	10000		70.3			106
Pergularia daemia (Forssk.) Chiov.	Ethyl acetate extract - leaves	Quinones	*Spodoptera litura* - L4	no choice	10000		71.82			106
Tylophora indica (Burm. f.) Merr.	Ethanolic extract	Unspecified	*Spodoptera litura* - L4		50000			8300		46
Tylophora indica (Burm. f.) Merr.	Ethanolic extract	Unspecified	*Spodoptera litura* - L5		50000			10100		46
Vincetoxicum hirundinaria Medik.	Plant extracts	Alkaloids, coumarins, phenols, terpenes and polyphenols	*Spodoptera littoralis* - L4	no choice		500	99.2		11	44
Araliaceae										
Panax ginseng C. A. Meyer	Stems and leaves extract - ginsenosides	Ginsenosides	*Plutella xylostella* - L2	choice	50000		87.82 ± 2.91	2740		47
Asteraceae										
Ageratina adenophora (Spreng.) R.M. King & H. Rob.	Hexane extract of aerial parts	Unspecified	*Crocidolomia pavonana* - L3	no choice	50000		87.3 ± 2.65	20600		107
Ageratina adenophora (Spreng.) R.M. King & H. Rob.	Hexane extract of aerial parts	Unspecified	*Plutella xylostella* - L3	no choice	50000		92.08 ± 3.6	14100		107
Ageratina adenophora (Spreng.) R.M. King & H. Rob.	Hexane extract of aerial parts	Unspecified	*Pieris brassicae* - L3	no choice	50000		81.67 ± 3.3	27700		107

Ageratum conyzoides L.	Young leaves hexane extract	Unspecified	*Plutella xylostella* - L3	choice	20000		100			108
Anthemis tinctoria L.	Plant extracts	Unspecified	*Leptinotarsa decemlineata* - L4	no choice		500	99.1		18	44
Artemisia abrotanum L.	Plant extracts	Unspecified	*Leptinotarsa decemlineata* - L4	no choice		500	91.8			44
Artemisia campestris L.	Plant extracts	Unspecified	*Leptinotarsa decemlineata* - L4	no choice		500	92.1			44
Artemisia capillaris Thunb.	2-acetonaphtone	Aromatic Carbonyl Compounds (*m*-Methoxyacetophenone; 2-Acetonaphthone)	*Pieris rapae crucivora* - L5	choice		68	99.2			109
Artemisia capillaris Thunb.	Methyleugenol	3,4-Dimethoxypropylbenzene; Methyleugenol	*Pieris rapae crucivora* - L5	choice			100 ± 3.87			110
Artemisia dracunculus L.	Dichloromethane, ethyl acetate and methanol extracts	Unspecified	*Epicauta atomaria* - A	choice		129	100			111
Balsamita major L.	Plant extracts	Unspecified	*Leptinotarsa decemlineata* - L4	no choice		500	98.5		13	44
Grindelia camporum Hook. & Arn.	Methanol extracts	Unspecified	*Leptinotarsa decemlineata* - L4	no choice		500	100		0.2	44
Chrysanthemum segetum L.	Crude methanolic extracts - flowers	Unspecified	*Spodoptera littoralis* - L6	choice	1000		78.55			112
Inula auriculata Boiss. & Balansa	Methanol extracts	Unspecified	*Leptinotarsa decemlineata* - L4	no choice		500	100		0.2	44
Jacobaea maritima (L.) Pelser & Meijden	Plant extracts	Unspecified	*Leptinotarsa decemlineata* - L4	no choice		500	92.8			44

Leuzea carthamoides (Willd.) DC.	Plant extracts	Unspecified	*Leptinotarsa decemlineata* - L4	no choice		500	100.0		58	44
Pyrethrum corymbosum (L.) Scop.	Methanol extracts	Unspecified	*Leptinotarsa decemlineata* - L4	no choice		500	100		3	44
Senecio fistulosus Poepp. ex DC	Furanoeremophilane	Eremophilane-type sesquiterpenes of the furanoeremophilane and eremophilanolide sesquiterpenes	*Spodoptera littoralis* - L6						0.64	48
Senecio kingii Hook.f.	Aerial parts alkaloidal extracts	Eremophilanolidess, shikimic acid derivatives, flavonoids	*Spodoptera littoralis* - L6	choice					0.09	49
Tagetes erecta L.	Leaf extracts	Tannins, terpenoids, phenols, saponins, flavonoids, alkaloids	*Spodoptera frugiperda* - L3	no choice	600		95			113
Tagetes patula L.	Ethyl acetate	Unspecified	Epicauta atomaria - A	choice		129	100			111
Tanacetum parthenium (L.) Sch.Bip.	Aerial parts - extract enriched with polar components	Camphor, *trans*-Chrysanthenyl acetate, *(Z)*-Spiroether	*Spodoptera littoralis* - L4	no choice					0.25 ul cm^{-2}	44
Tithonia diversifolia (Hemsl.) A.Gray	Leaves extract	Tagitin C	*Helicoverpa armigera* - L3	choice	100000		100			114
Xeranthemum cylindraceum Sibth. & Sm.	Methanol extracts	Unspecified	*Leptinotarsa decemlineata* - L4	no choice		500	96.3		8	44
Boraginaceae										
Echium wildpretii H. Pearson ex Hook. f. subsp. *wildpretii*	Fraction 2 from ethanol extract: hexane/ethyl acetate, 90 : 10 v/v); steroidal fraction - compound 3	Fatty Acid Esters and Phytosterols	*Leptinotarsa decemlineata* (unspecified)	choice					0.40	50

Buxaceae										
Sarcococca brevifolia (Müll.Arg.) Stapf ex Gamble	Aerial parts - alkaloid	Alkaloids	*Epilachna varivestis* - L4	choice	2500		100			115
Caricaceae										
Carica papaya L.	Leaf extracts	Alkaloid compounds, phenols, flavonoids	*Spodoptera litura* - L3	choice	400000		95.7			116
Convolvulaceae										
Convolvulus lineatus L.	Plant extracts	Unspecified	*Leptinotarsa decemlineata* - L4	no choice		500	97.4		143	44
Crassulaceae										
Kalanchoe daigremontiana Hamet & Perrier	Polar extract - leaves	Triterpenoid, alkaloid	*Plutella xylostella - L4*	no choice	12500		84.85			117
Sedum rosea (L.) Scop.	Plant extracts	Unspecified	*Leptinotarsa decemlineata* - L4	no choice		500	87.9			44
Fabaceae										
Astragalus glycyphyllos L.	Plant extracts	Unspecified	*Leptinotarsa decemlineata* - L4	no choice		500	89.5			44
Caesalpinia bonduc (L.) Roxb.	Chloroform extract – fraction 3	Coumarins, flavonoids, terpenoids, phenols, quinones	*Helicoverpa armigera* - L3	no choice				357.13		51
Melilotus albus Medik.	Plant extracts	Unspecified	*Leptinotarsa decemlineata* - L4	no choice		500	100.0		108	44
Millettia pachycarpa (Benth.)	Fresh leaves methanol extract -> dichlormethane - > fraction 2	Triterpenoid (lupeol)	*Spodoptera litura* - L3	no choice				227.13		52

Psoralea bituminosa (L.) Stirton	Plant extracts	Unspecified	*Spodoptera littoralis*	no choice		500	96.8		72	44
Psoralea bituminosa (L.) Stirton	Plant extracts	Unspecified	*Leptinotarsa decemlineata* - L4	no choice		500	100		116	44
Trifolium arvense L.	Plant extracts	Unspecified	*Leptinotarsa decemlineata* - L4	no choice		500	100		15	44
Hypericaceae										
Hypericum perforatum L.	Plant extracts	Unspecified	*Leptinotarsa decemlineata* - L4	no choice		500	98.5		94	44
Lamiaceae										
Lavandula dentata L.	Methanol extract	Unspecified	*Epicauta atomaria* - A	choice		129	100			111
Salvia glutinosa L.	Plant extracts	Unspecified	*Leptinotarsa decemlineata* - L4	no choice		500	100		115	44
Stachys byzantina K. Koch ex Scheele	Plant extracts	Unspecified	*Leptinotarsa decemlineata* - L4	no choice		500	100		18	44
Teucrium hircanicum L.	Methanol extracts	Unspecified	*Leptinotarsa decemlineata* - L4	no choice		500	100		6	44
Teucrium chamaedrys L.	Plant extracts	Unspecified	*Leptinotarsa decemlineata* - L4	no choice		500	94.1			44
Thymus fragrantissimus Samen.	Plant extracts	Unspecified	*Leptinotarsa decemlineata* - L4	no choice		500	93.5			44
Thymus serpyllum L.	Plant extracts	Unspecified	*Leptinotarsa decemlineata* - L4	no choice		500	100		15	44
Thymus vulgaris L.	Ethyl acetate extract	Unspecified	*Epicauta atomaria* - A	choice		129	100			111
Lauraceae										

Persea indica (L.) Spreng.	Stem extract	Ryanoids	*Spodoptera littoralis* - L6	no choice		100			8.5	53
Loganiaceae										
Strychnos nux-vomica L.	MeOH bark	Unspecified	*Epilachna varivestis* - L4	choice	2500		100			115
Meliaceae										
Aglaia elaeagnoidea Benth.	Aerial parts – chloroform extract – fraction 6K	Mixture of limonoids	*Spodoptera litura* - L5	choice		10 and 20	80			118
Azadirachta indica A.Juss	Aqueous neem oil	Azadirachtin	*Caliroa cerasi* - L	choice	20000		> 90		41.4	119
Azadirachta indica A.Juss	Aqueous neem oil	Azadirachtin	*Caliroa cerasi* - L	no choice	40000		> 90		93.7	119
Azadirachta indica A.Juss	Oil sample SCR/50/3	Azadirachtin	*Peridroma saucia* - L5	choice	4026				2	40
Melia volkensii Gürke	Refined seed extract	Terpenoids	*Epilachna varivestis* - A	choice					2.3	54
Trichilia americana (Sessé & Moc.) T.D.Penn.	Crude methanolic extract of wood	Unspecified	*Spodoptera litura* - L5	choice a no choice		0.5 and 5	> 90			120
Menispermaceae										
Diploclisia glaucescens (Blume) Diels	Stem - CH2Cl2	Unspecified	*Epilachna varivestis* - L4	choice	2500		100			115
Coscinium fenestratum (Gaertn.) Colebr.	Stem - meoh	Unspecified	*Epilachna varivestis* - L4	choice	2500		100			115
Moringaceae										

Moringa oleifera Lam., 1783	Leaves extracts - ethanol	Unspecified	*Spodoptera litura* - L4	choice	125000		79.76			121
Myrtaceae										
Syzygium caryophyllum (L.) Alston	Stem - MeOH	Unspecified	*Epilachna varivestis* - L4	choice	2500		100			115
Picramniaceae										
Picramnia sellowii Planch.	Leaf; wood extracts	Anthraquinones, chlorogenic acids	*Epilachna paenulata* - A	choice		100000	95; 95			122
Piperaceae										
Piper nigrum L.	Leaves extracts - ethanol	Alkaloids	*Spodoptera litura* - L4	no choice	125000		85.57			121
Piper ribesioides Wall.	Ethyl acetate extract - twigs	Piperine, methyl piperate, *n*-cinnamoyl-(2-phenylethyl) amine, lemairamin, *n,n*-diphenyl cinnamide, cinnamic acid, methyl cinnamate, pinostobrin, pinocembrin	*Spodoptera exigua* - L3	choice	500–10000		81.8		26.25	123
Polemoniaceae										
Gilia capitata Sims	Plant extracts	Unspecified	*Leptinotarsa decemlineata* - L4	no choice		500	97.9		125	44
Polygalaceae										
Polygala comosa Schkuhr	Plant extracts	Unspecified	*Leptinotarsa decemlineata* - L4	no choice		500	98.3		295	44
Ranunculaceae										

Consolida ajacis (L.) Schur	Ethyl acetate seed extract	Ethyl linoleate	*Plutella xylostella* - L3	choice		5.68 ul cm^{-2}	> 90			124
Resedaceae										
Reseda odorata L.	Plant extracts	Unspecified	*Leptinotarsa decemlineata* - L4	no choice		500	90.6			44
Rubiaceae										
Catunaregam spinosa (Thunb.) Tirveng.	Fruit methanolic extract; n-butanol extract	Triterpenoids	*Plutella xylostella* - L3	no choice				1790 and 1330		125
Catunaregam spinosa (Thunb.) Tirveng.	Fruit methanolic extract; n-butanol extract	Triterpenoids	*Pieris rapae* - L4	no choice				3840 and 2170		125
Rutaceae										
Atalantia monophylla (Roxb.) A. DC.	Hexane extract	Alkaloids, coumarin, quinone and terpenoids	*Helicoverpa armigera* - L3	no choice	50000		79.06 ± 3.42			126
Clausena anisata (Willd.) Hook.f. ex Benth.	Chloroform root extracts	Osthol (coumarin derivate)	*Helicoverpa armigera* - L5	choice	20000			140		55
Clausena anisata (Willd.) Hook.f. ex Benth.	Petroleum ether root extracts	Osthol (coumarin derivate)	*Helicoverpa armigera* - L5	choice	20000			160		55
Feronia limonia Rutaceae (L.) Swingle	Bark – methanol extract	Unspecified	*Epilachna varivestis* - L4	choice	2500		100			115
Murraya paniculata (L.) Jack	Leaves extracts - ethanol	Unspecified	*Spodoptera litura* - L4	no choice/choice	125000		98.9 / 83.64			121
Scrophulariaceae										

Scrophularia nodosa L.	Plant extracts	Unspecified	*Leptinotarsa decemlineata* - L4	no choice		500	100.0		37	44
Simaroubaceae										
Castela coccinea Griseb.	Leaf extracts	Alkaloids, steroids	*Epilachna paenulata* - A	choice		100000	97			122
Solanaceae										
Solanum xanthocarpum Schrad. & Wendl.	Chloroform extract, fraction 4	Terpenoids, flavonoid, and quinone	*Helicoverpa armigera - L3*	no choice	1000		79.57	378.30		56
Verbenaceae										
Lantana camara L.	Leaf extract - ethyl acetate fraction	Alkaloids, saponins, steroids and triterpenoids	*Crocidolomia pavonana* - L4	no choice	3000		80			127
Lippia alba (P. Mill.) N.E. Br. ex Britt. & P. Wilson	Leaves extracts	Flavones, glycosides, glucuronides	*Spodoptera frugiperda* - L2	no choice	5300		87			128
Verbena officinalis L.	Plant extracts	Unspecified	*Leptinotarsa decemlineata* - L4	no choice		500	100		17	44
Vitaceae										
Vitis vinifera L.	Vine-shoot wastes: Conventional Solid-Liquid Extraction 60 min	Flavanols	*Leptinotarsa decemlineata* - A	choice		100			0.08	57
Winteraceae										
Drimys winteri J.R. Forster et G. Forster	Drimane polygodial	Drimane sesquiterpenoids	*Spodoptera littoralis* - L6	choice		1000	94.7			129

Table S2. Antifeedant efficacy of plant essential oils (full version with FDI > 70%); Insect: L = larvae (instar) and A = adults; The concentration is given in μg mL^{-1} for liquids, or in μg cm^{-2} for the dose of active ingredient calculated on a given leaf surface area, the resulting value also correspods to this: EC_{50} for liquids, or ED_{50} for solids.

Family/Plant	Parts	Majority compounds with antifeedant effect	Insect (instrar)	Test	Concentration (μg mL^{-1})	Dose (μg cm^{-2})	FDI (%) ± SE	EC_{50} (μg mL^{-1})	ED_{50} (μg cm^{-2}) ± SE	References
Acoraceae										
Acorus calamus L.	Rhizomes - cis asarone	Cis-asarone, trans-asarone	*Peridroma saucia* - L4	choice		27	100		2.5	58
Acorus calamus L.	Hydrodistillation	1,4-cineole, camphor, β-pinene, cis-verbenol, β-caryophyllene.	*Plutella xylostella* - L3	choice		15000		100		130
Apiaceae										
Angelica archangelica L.	Seeds	β-Phellandrene, sabinene, α-pinene, α-phellandrene	*Spodoptera littoralis* - L3	no choice					7.12	45
Asteraceae										
Ageratina adenophora (Spreng.) King & H.Rob.	Twig with leaves	Γ-muurolene, o-cymene, bornyl acetate, α-bisabolol	*Plutella xylostella* - L3	no choice		0.6 ul cm^{-2}	87.46		0.26 ul cm^{-2}	107
Artemisia absinthium L.	Aerial parts	(z)-2,6-dimethylocta-5,7-dien-2,3-diol, chrysanthenol, diterpene c20h28o, c10h18o sesquiterpene	*Spodoptera littoralis* - L6	choice		100	89 ± 3			131
Artemisia annua L.	Rainy and winter season EO	Camphor, germacrene-D, β-caryophyllene, eucalyptol,	*Spodoptera litura* - L2	no choice	8 uL mL^{-1}		90.89 and 87.92			132
Artemisia herba-alba Asso	Leaves, flowers	Camphor, α-thujone, β-thujone, 1,8-cineole	*Spodoptera littoralis* - L6	choice		100	93.7 ± 2.2		14.6	61
Artemisia nakaii Pamp.	Aerial parts	Feropodin, (+)-camphor, 1,8-cineole, rishitin	*Spodoptera litura* - L3	choice					3.76 ± 0.73	59
Artemisia pedemontana subsp. assoana (Willk.) Rivas Mart. *(greenhouse)*	Aerial parts	Camphor, 1,8-cineole, terpinen-4-ol, borneol	*Spodoptera littoralis* - L6	choice		100	85.6 ± 7.9			133

Artemisia scoparia Maxim.	Aerial parts	β-pinene, limonene, α-terpinolene, capilene	*Spodoptera litura* - L3	no choice			66.91	3.6 ul mL^{-1}		134
Anacardiaceae										
Cotinus coggygria Scop.	EO from aerial parts	α-pinene, limonene, β-myrcen, α-terpinolene, β-caryophyllene	*Spilosoma obliqua* - L3	no choice			97.4			135
Geraniaceae										
Geranium macrorrhizum L. (GH)	Aerial parts	β-elemenone, thymol, germacrone, nerolidol acetate	*Spodoptera littoralis* - L6	choice		100	87.8 ± 9.7		22 (14–34)	98
Pelargonium graveolens (Thunb.) L'Hér.	Hydrodistillation	Geraniol, linalool, and citronellol	*Plutella xylostella* - L3	choice	10000		100			130
Lamiaceae										
Hyssopus officinalis L.	Aerial parts	1,8-Cineole, β-pinene, pinocarvone, sabinene	*Spodoptera littoralis* - L6	choice		100	78.99 ± 8.1		49 (31 – 67)	136
Lavandula luisieri (Rozeira) - cultivated pop.	Flowering parts	3-Oxo-cadinol, 2,3,4,4-Tetramethyl-5-methylidenecyclopent-2-en-1-one, Hydroxymethyl-2,3,4,4-tetramethylcyclopent-2-en-1-one	*Spodoptera littoralis* - L6						10.23	60
Lavandula stoechas subsp. luisieri Rozeira (Portuguese population, Casal da Fraga)	Fresh leaves	Trans-α-necrodyl acetate, trans-α-necrodol, sesquiterpene acetate $C_{17}H_{28}O_3$, viridiflorol	*Spodoptera littoralis* - L6	choice		100	73 ± 10			137

Lavandula stoechas subsp. luisieri Rozeira (Portuguese population, Castelo Branco 2006)	Leaves	Trans-α-Necrodyl acetate, p-cymene, cis-α-necrodyl acetate, linalool	*Spodoptera littoralis* - L6	choice		100	90 ± 3			137
Lavandula stoechas subsp. luisieri Rozeira (Portuguese population, Castelo Branco 2007)	Leaves	Trans-α-Necrodyl acetate, fenchone, cis-α-necrodyl acetate, camphor	*Spodoptera littoralis* - L6	choice		100	97 ± 1			137
Lavandula stoechas subsp. luisieri Rozeira (Portuguese population, Castelo Branco 2007)	Flowers	Camphor, 2,3,4,4-tetramethyl-5-methylen-2-cyclopenten-1-one, 1,8-cineole, bornyl acetate	*Spodoptera littoralis* - L6	choice		100	90 ± 7			137
Lavandula stoechas subsp. luisieri Rozeira (Portuguese population, Penamacor)	Leaves	Trans-α-Necrodyl acetate, β-selinene, trans-α-necrodol, fenchone	*Spodoptera littoralis* - L6	choice		100	96 ± 2			137
Lavandula stoechas subsp. luisieri Rozeira (Portuguese population, Penamacor)	Flowers	Trans-α-Necrodyl acetate, β-selinene, trans-α-necrodol, fenchone	*Spodoptera littoralis* - L6	choice		100	97 ± 1			137
Lavandula x intermedia var. Super Emeric ex Loisel.	Aerial parts	Linalyl acetate, linalool, camphor, borneol	*Spodoptera littoralis* - L6	choice		100	89.11 ± 6.7		25 (22 – 28)	132
Mentha crispa Ten.	commercial EO	Camphene, D-carvone	*Trichopulsia ni* - L3	choice		50	83.6		24	138
Mentha pulegium L. (Orestiada - Pul1)	Leaves and flowers	Piperitone, limonene, 3-octanol, menthone	*Spodoptera littoralis* - L6	choice		100	80.8 ± 4.6			99

Mentha pulegium L. (Orestiada - Pul1)	Leaves and flowers	Pulegone, isomenthone, piperitenone, menthone	*Spodoptera littoralis* - L6	choice		100	75.1 ± 2.7			99
Mentha pulegium L.	Leaves and flowers	Pulegone, 1,3,4-trimethyl-3-cyclohexene-1-carboxaldehyde, piperitenone	*Spodoptera littoralis* - L6	choice		100	100.0 ± 0.0		1.3 (0.4, 4.1)	61
Nepeta hindostana (B. Heyne ex Roth) Haines	Aerial parts - EO	β-caryophyllene, *trans*-β-farnesene, *m*-camphorene, *p*-camphorene	*Spilosoma obliqua* - L4	no choice			98.3			139
Nepeta hindostana (B. Heyne ex Roth) Haines	Roots - EO	(*Z,Z*)-heptadeca-1,8,11-triene; methyl linoleate; linoleic acid; stigmast-5-en-3-ol, oleate	*Spilosoma obliqua* - L4	no choice			98.1			139
Ocimum basilicum L.	EO from leaves	D-linalool and (+/-) linalool	*Lymantria dispar* - L2	choice	1000		92.71			140
Ocimum basilicum L. (eugenol-rich type)	Hydrodistillation	Eugenol	*Plutella xylostella* - L3	choice	10000		100			130
Pogostemon cablin (Blanco) Benth.	Leaves	Patchouli alcohol, α-bulnesene, α-guaiene	*Spodoptera litura* - L3	no choice	500		91.3 ± 1.0			141
Satureja hortensis L.	Aerial parts - EO	γ-terpinene, carvacrol	*Leptinotarsa decemlineata* - L4	no choice	2000; 20000		87.45 and 100			142
Satureja montana L.	Aerial parts at the flowering stage	Carvacrol	*Spodoptera littoralis* - L6	choice		100			39.5	143
Thymus linearis Benth.	Aerial parts - witer and rainy season	Thymol, γ-terpinene	*Spodoptera litura* - L2	no choice	8 uL mL^{-1}		98.91 and 95.8			144
Thymus vulgaris L.	Aerial parts - EO	p-cymene, γ-terpinene, thymol	*Leptinotarsa decemlineata* - L4	no choice	2000; 20000		76.22 and 100			142
Lauraceae										

Cinnamomum zeylanicum Blume	Commercial EO	*(E)*-cinnamaldehyde, camphene	*Trichopulsia ni* - L3	choice		50	91.5		12.5	138
Myrtaceae										
Melaleuca alternifolia (Maiden & Betche) Chee	Commercial EO	Terpinen-4-ol, γ-terpinene, α-terpineol, α-terpineol, 1,8-cineole	*Helicoverpa armigera* - L3	no choice	40000		97.8 ± 2.88	8930		145
Syzygium aromaticum (L.) Merr. & L.M. Perry	Leaves	Eugenol, β-caryophyllene,	*Plutella xylostella* - L3: susceptible a resistant pop. to deltamethrin	choice				14.97 and 26.74		92
Syzygium aromaticum (L.) Merr. & L.M. Perry	Commercial EO	Eugenol, *(E)*-isoeugenol	*Trichopulsia n*i - L3	choice		50	78.6		16.4	138
Piperaceae										
Piper divaricatum G.Mey.	Leaves	Eugenol, methyl eugenol, γ-elemene, asarone	*Spodoptera littoralis* - L6	choice		100	70.7 ± 5.2			63
Piper hispidinervum C.DC.	Fresh leaves and twigs	Safrole, terpinolene	*Leptinotarsa decemlineata* - A	choice					0.4	62
Piper hispidinervum C.DC.	Fresh leaves and twigs	Safrole	*Spodoptera littoralis* - L	choice					3.1	62
Piper hispidum Sw.	Leaves	Limonene, δ-3-carene, p-cymene, elemol, spathulenol	*Spodoptera littoralis* - L6	choice		100	76.2 ± 2.5		48.0 (47.8-48.2)	63
Piper marginatum Jacq. (Turbaco)	Leaves	Germacrene D, b-elemene, germacren-D-4-ol	*Spodoptera littoralis* - L6	choice		10	80.6±12.1			146
Piper sanctifelicis Trel.	Leaves	δ-3-carene, limonene, p-cymene, β-pinene, nerolidol	*Spodoptera littoralis* - L6	choice		10	96.9 ± 0.9		4.5 (4.3-4.7)	63
Poaceae										
Cymbopogon citratus (DC.) Stapf	Commercial EO	Citral, limonene	*Spodoptera litura* - L3	no choice				59410		147

Cymbopogon citratus (DC.) Stapf	Sun-dried lemon grass leaves - stem distillation	Unspecified	*Spodoptera exigua* - L3	choice	20000		100			148
Cymbopogon nardus L.	Crude oil from leaves	Citronellal	*Crocidolomia pavanana* - L3	no choice	1000		98.38			149
Rutaceae										
Citrus aurantifolia (L.) Swingle	EO a limonene	γ-Muurolene, o-cymene, bornyl acetate, α-bisabolol	*Plutella xylostella* - L3 - deltamethrin susceptible strain	choice				68.93		64
Citrus aurantiifolia (L.) Swingle	Commercial EO	γ-Muurolene, o-cymene, bornyl acetate, α-bisabolol	*Plutella xylostella* - L3 - deltamethrin resistant strain	choice				180.9		64
Citrus limon (L.) Burm. f.	Commercial EO	γ-Muurolene, o-cymene, bornyl acetate, α-bisabolol	*Plutella xylostella* - L3 - deltamethrin resistant strain	choice				181.3		64
Zingiberaceae										
Zingiber officinale Roscoe	Fresh rhizomes -> steam distillation -> ginger oleoresin	Curcumene	*Spilosoma obliqua* - L3	no choice	10000		82	3100		150

Table S3. Antifeedant efficacy of isolated compounds (full version with FDI > 70%); Insect: L = larvae (instar) and A = adults; The concentration is given in μg mL^{-1} for liquids, or in μg cm^{-2} for the dose of active ingredient calculated on a given leaf surface area, the resulting value also correspods to this: EC_{50} for liquids, or ED_{50} for solids.

Plant	Type	Insect (instar)	test	Concentration (μg mL^{-1})	Dose (μg cm^{-2})	FDI (%) ± SE	EC_{50} (μg mL^{-1})	ED_{50} (μg cm^{-2})	References
Araliaceae									
Panax ginseng C. A. Meyer	Total ginsenosides	*Pieris rapae - L3*	choice	20000 and 10000		86.09 and 80.64			151
Panax ginseng C. A.Meyer	Panaxadiols saponins	*Ostrinia furnacalis - L3*	choice	100000		80.9	7060		152
Asteraceae									
Artemisia vestita Wall. ex Besser	Dihydroestafiatone	*Plutella xylostella* - L3	no choice					25.3	153
Carpesium abrotanoides L.	Air-dried fruits - compound 1	*Plutella xylostella* - L3	choice				19.84		65
Eupatorium adenophorum Spreng	Sesquiterpenoids, compound 2 and 3	*Helicoverpa armigera* - L2	choice					2.57 and 3.04	66
Flourensia oolepis S.F. Blake	Aerial parts - flavonoid pinocembrin	*Epilachna paenulata* - L3	choice		50	98		10.08	67
Pericallis spp.	3-ethoxy-hydroxy-tremetone; (-)-eupachinin A	*Spodoptera litoralis* - L6	choice				130; 1040		68
Senecio adenotrichius DC.	Compound 1 - dehydrofukinone,	*Spodoptera litoralis* - L6	unspecified					1.68	69
Senecio palmensis C. Sm.	11α-Acetoxy-5-(angeloyloxy)silphinen-3-one	*Leptinotarsa decemlineata* - L4	choice - short 6 h					1.69	70
Senecio palmensis C. Sm.	11*β*,5α- dihydroxysilphinen- 3-one	*Leptinotarsa decemlineata* - L4	no choice					11.32	70
Smallanthus sonchifolius (Poepp. & Endl.) H. Rob	Uvedalin	*Spodoptera litura* - L3	choice					8	71
Brassicaceae									
Iberis amara L.	Seeds: 2-*O*-*β*-D-glucopyranosyl cucurbitacin E	*Pieris rapae* - L3	choice	200		84.7			154

Colchicaceae									
Gloriosa superba L.	Chloroform tuber extract - GST4	*Spodoptera litura* - L3	no choice				26		72
Cucurbitaceae									
Citrullus colocynthis (L.) Schrad.	Cucurbitacin E - fruits (fraction III)	*Spodoptera litura* - L5	choice	50		81.2	24.1		73
Cupressaceae									
Juniperus sabina L.	Petroleum ether extract - deoxypodophyllotoxin (1)	*Pieris rapae* - L5	unspecified				60 (48 hrs)		74
Cyperaceae									
Cyperus radians Nees & Meyen ex Kunth	3´,4,4´,6 - tetramethoxyaurone	*Spodoptera litura* - L3	choice					41.08	155
Droseraceae									
Dionaea muscipula Sol. ex J. Ellis	Plumbagin	*Spodoptera litura* - L3	choice	5000		100			156
Ericaceae									
Pieris formosa (Wallich) D. Don	Grayanane diterpenoids - 10	*Spodoptera exigua*	choice modified					6.58	75
Pieris japonica (Thunb.) D. Don ex G. Don	Neopierisoid B - isolated from flowers	*Pieris brassicae* - L3	choice					5.33	76
Pieris japonica (Thunb.) D. Don ex G. Don	Flower diterpenoids - C10	*Pieris brassicae* - L3	choice					0.03	77
Rhododendron molle (Blume) G. Don	Rhodojaponin III - grayanoid diterpene from flowers	*Pieris rapae* - L3	no choice				1.16		78
Rhododendron molle (Blume) G. Don	Rhodojaponin III - grayanoid diterpene from flowers	*Pieris rapae - L5*	no choice				15.85		78
Euphorbiaceae									
Croton jatrophoides Pax.	Limonoids dumnin, dumsenin	*Pectinophora gossypiella* - L2, *Spodoptera frugiperda* - L2	choice				≤ 2		79

Croton jatrophoides Pax.	Limonoids from methanol extract - Musidunin	*Pectinophora gossypiella* - L2	choice				3		80
Croton jatrophoides Pax.	Limonoids from methanol extract - Musiduol	*Pectinophora gossypiella* - L2	choice				4		80
Croton jatrophoides Pax.	Limonoids from methanol extract - Musiduol	*Spodoptera frugiperda* - L2	choice				2		80
Euphorbia fischeriana Steud.	Latex diterpenoids - 1	*Helicoverpa armigera* – L (unspecified)	choice					2.59	157
Euphorbia paralias L.	Ursane type triterpenoid - compound 21 (uvaol)	*Leptinotarsa decemlineata* - A	choice					0.2	81
Euphorbia paralias L.	Ursane type triterpenoid - compound 21 (uvaol)	*Spodoptera litoralis* - L6	choice					3.3	81
Fabaceae									
Cassia fistula L.	Rhein	*Helicoverpa armigera* – L4	no choice	1000		76.13			158
Glycine max (L.) Merr.	Glyceolin - isoflavonoid	*Epilachna varivestis* - A	choice						159
Glycine max (L.) Merr.	Glyceolin - isoflavonoid	*Diabrotica undecimpunctata* - A	choice						159
Pterocarpus macrocarpus Kurz	Homopterocarpin	*Spodoptera litura* - L3	choice					0.04	82
Lamiaceae									
Clerodendrum infortunatum L.	Clerodane diterpenoids - compound 1	*Helicoverpa armigera* - L3	choice				6		83
Hyssopus cuspidatus Boriss.	Ursolic acid	*Plutella xylostella* - L3	no choice					25.47	160
Leucosceptrum canum Sm.	Sesterpenoid - compound 1	*Spodoptera exigua* – L (unspecified)	unspecified						161
Mentha suaveolens Ehrh.	Piperitenone	*Spodoptera littoralis* - L6	choice		100				144
Mentha suaveolens Ehrh.	Piperitenone oxide	*Spodoptera littoralis* - L6	choice		100				144
Scutellaria baicalensis Georgi	6-methylflavone	*Spodoptera litura*	choice					8.27	162
Scutellaria L. - 2 species	Jodrellin B	*Spodoptera litoralis* - L6	choice	100		100			163

Scutellaria L. - 2 species	Scutecyprol B	*Spodoptera litoralis* - L6	choice	100		100			163
Scutellaria L. - 2 species	Jodrelin B	*Spodoptera frugiperda* - L6	choice	100		98±0.9			163
Scutellaria L. - 2 species	Jodrelin B	*Mamestra brassicae* - L6	choice	100		95 ± 1.2			163
Scutellaria L. - 2 species	Jodrelin B	*Pieris brassicae* - L5	choice	100		96 ± 0.9			163
Scutellaria L. - 2 species	Jodrelin B	*Helicoverpa armigera* - L6	choice	100		90 ± 2.4			163
Scutellaria L.	Jodrellin B	*Spodoptera litoralis* – L (unspecified)	choice	100		100			164
Scutellaria L.	Dihydroclerodin	*Spodoptera litoralis* – L (unspecified)	choice	100		95			164
Scutellaria L.	Scutecyprol B	*Spodoptera litoralis* – L (unspecified)	choice	100		100			164
Scutellaria L.	Scutalpin C	*Spodoptera litoralis* – L (unspecified)	choice	100		97			164
Scutellaria L.	Dihydroclerodin	*Spodoptera exempta* – L (unspecified)	choice	100		94			164
Teucrium massiliense L.	Ajugarin semisynthetic derivatives - teumassilenins A (11)	*Leptinotarsa decemlineata* - L4	no choice	1000		75.62			165
Teucrium massiliense L.	Ajugarin semisynthetic derivatives - teumassilenins A (11)	*Leptinotarsa decemlineata* - L4	choice	1000		88.19			165
Teucrium massiliense L.	Ajugarin semisynthetic derivatives - Ajugarin I (1)	*Spodoptera exigua* - L5	no choice	1000		70.04			165
Teucrium massiliense L.	Ajugarin semisynthetic derivatives - chlorohydrin (8)	*Spodoptera exigua* - L5	choice	1000		91.36			165
Lauraceae									
Persea indica (L.) Spreng.	Anhydrocinnzeylanine	*Spodoptera littoralis* - L5	choice					0.09	84

Persea indica (L.) Spreng.	Anhydrocinnzeylanine	*Leptinotarsa decemlineata* - A	choice					0.94	84
Persea indica (L.) Spreng.	Cinnzeylanine	*Spodoptera litoralis* – L5	choice		2			0.004	85
Persea indica (L.) Spreng.	Cinnzeylanone	*Leptinotarsa decemlineata* - A	choice		2			0.08	85
Meliaceae									
Aglaia odorata Lour.	Rocaglamide from dried twigs	*Peridroma saucia - L4*	choice					3.45	86
Aphanamixis polystachya (Wall.) R.Parker	Limonoids from leaves and twigs extracts - Aphanamixoid F (4)	*Helicoverpa armigera* – L (unspecified)	choice	2000		> 70			166
Azadirachta indica A. Juss.	Azadirachtin	*Cnaphalocrocis medinalis* - L5	choice	2		93.2 ± 7.6			167
Azadirachta indica A. Juss.	Azadirachtin	*Ostrinia nubilalis* - L1 and L3	no choice				3.5 and 24		87
Azadirachta indica A. Juss.	Toosedanin	*Pieris brassicae* - L5	choice			> 90			168
Cedrela toona Roxb., *Cedrela odorata* Ruiz & Pav.	Cedrelone	*Mamestra configurata* - L4	choice		28.6	75.9		14.90	169
Melia volkensii Gürke	Volkensin	*Spodoptera frugiperda* - L3	choice					3.5	88
Melia volkensii Gürke	Xanthotoxin 99%	*Trichopulsia ni* - L3	choice					0.9	54
Melia toosendan Siebold & Zucc.	Toosedanin - limonoid isolated from bark	*Peridroma saucia* - L4	choice					8.04	89
Swietenia macrophylla King, *S. aubrevilleana* Stehlé & Cusin	Limonoid - swietenolide	*Spodoptera frugiperda* - L6	unspecified	1000		94.1 ± 2.9			170
Piperaceae									
Piper ribesioides Wall.	Pinostrobin - isolated from twigs	*Spodoptera exigua* - L3	choice			90		14.39	123
Piper ribesioides Wall.	Piperine	*Spodoptera litura* - L3	choice		3.18 - 318,5			3.1	90
Plantaginaceae									

Linaria saxatilis (L.) Chaz.	Neo-clerodane diterpenoids, compound 2	*Leptinotarsa decemlineata* - A	choice / no choice					10.5 / 8.5	91
Linaria saxatilis (L.) Chaz.	Neo-clerodane diterpenoids, compound 6	*Leptinotarsa decemlineata* - A	choice / no choice					12.8 / 7.7	91
Linaria saxatilis (L.) Chaz.	Neo-clerodane diterpenoids, compound 8	*Leptinotarsa decemlineata* - A	choice					6.4	91
Ranunculaceae									
Aconitum leucostomum Vorosch.	Anthranoyllycoctonine and avadharidine	*Spodoptera exigua* - L3	choice		4.78 ul cm^{-2}			730; 840	77
Aconitum leucostomum Vorosch.	Diterpenoids - compound 2	*Spodoptera exigua* – L (unspecified)						1540	171
Aconitum rockii H. R. Fletcher & Lauener	14-benzoylneoline	*Spodoptera exigua* - L1	choice					130	172
Aconitum spp.	Chasmanthinine	*Spodoptera exigua* - L3	choice	62500–1000000				70	173
Clematis aethusifolia Turcz	Triterpenoid saponins - monodesmosides 1, 2, 5 (from n-buoh extract)	*Plutella xylostella* - L3	choice	500–1500			733.67–844.77		174
Coptis japonica Makino	Berberine chloride + palmatine iodide (1:1)	*Hyphantria cunea* - L4	no choice	250 and 500		82.3 and 100			175
Coptis japonica Makino	Berberine chloride	*Agelastica coerulea* - A	no choice	250 and 500		91.1 and 97.2			175
Delphinium naviculare var. *lasiocarpum* W. T. Wang	Shawuresine	*Spodoptera exigua* - L3	choice; no choice					420; 810	176
Rubiaceae									
Catunaregam spinosa (Thunb.) Tirveng.	Triterpenoid saponins - swartziatrioside	*Plutella xylostella* - L2	no choice					106.73	177

Rubia akane Nakai	Anthraquinone aldehyde nordamnacanthal	*Spodoptera litura* - L3	choice		83			32.19	178
Rutaceae									
Citrus aurantiifolia (Christm.) Swingle	Limonene	*Plutella xylostella* - L3 - deltamethrin susceptible strain	choice				4.44		92
Citrus aurantiifolia (Christm.) Swingle	Limonene	*Plutella xylostella* - L3 - deltamethrin resistant strain	choice				17.83		92
Salvadoraceae									
Azima tetracantha Lam.	Friedelin	*Helicoverpa armigera* - L3	no choice	1000			130.47		179
Azima tetracantha Lam.	Friedelin	*Spodoptera litura* - L3	no choice	1000			226.41		179
Sapindaceae									
Dodonaea viscosa (L.) Jacq.	Total saponins	*Spodoptera litura* - L4	no choice				1621.81		180
Sapotaceae									
Diploknema butyracea (Roxb.) H.J.Lam	Alkaline hydrolyzed saponin	*Spodoptera litura* - L3	choice	10000		81.8	1200		181
Simaroubaceae									
Eurycoma longifolia Jack	Eurycomanone	*Plutella xylostella* - L3	choice	150			14.2		93
Solanaceae									
Duboisia myoporoides R.Br.	Ursolic acid	*Spilosoma obligua, Spodoptera litura* (L – unspecified)		5000			1730 and 1986		182
Solanum melongena L.	Fruits -caffeic acid methyl ester	*Spodoptera litura* - L3	no choice		100	100		52.02	95
Solanum melongena L.	Fruits -caffeic acid methyl ester	*Achaea janata* - L3	no choice		100	100		41.02	95
Vitaceae									
Vitis vinifera L.	Stilbenes from roots (vitisin A, vitisin B)	*Leptinotarsa decemlineata* - L2	no choice		100	92.3 and 91.6		23.7 and 34.5	183

Winteraceae									
Drimys winteri J.R. Forster et G. Forster	Polygodial	*Spodoptera frugiperda* - L3	choice		50			5.59	94
Unspecified									
	α-Pinene	*Spodoptera litura* - L3	no choice					1.13 (0.65−1.78) uL cm^{-2}	95
	β-Caryophyllene	*Spodoptera littoralis* - L6	choice		50	90.7		26.2 (21.7, 31.6)	100
	β-Caryophyllene	*Spodoptera littoralis* - L6	choice		50	91		26.2 (21.7, 31.6)	184
	Aconitine	*Diabrotica virgifera* - A	choice					0.267	96
	Carvacrol	*Spodoptera littoralis* - L4	no choice		250	100			185
	Dehydrofukinone (SO)	*Spodoptera littoralis* - L6	choice		50	95 ± 1		1.68 (1.38 – 2.04)	69
	Derivatives of eugenol and thymol (6)	*Plutella xylostella* - L3	choice				4.29		97
	Derivatives of eugenol and thymol (8)	*Plutella xylostella* - L3	choice				3.3		97
	Derivatives of eugenol and thymol (10)	*Plutella xylostella* - L3	choice				6.52		97
	Derivatives of eugenol and thymol (thymol)	*Plutella xylostella* - L3	choice				6.38		97
	Drimane compounds: dialdehyde (5) - polygodial	*Leptinotarsa decemlineata* - L4	choice			92 ± 0.02			186
	Eugenol	*Spodoptera littoralis* - L4	no choice		250	100			185
	GABA/ glycinergic antagonists - quinine, strychnine	*Diabrotica virgifera* - A	choice						187
	GABA/ glycinergic antagonists - brucine	*Diabrotica berberi* - A	choice						187

	Geijerene	*Spodoptera littoralis* - L3	choice		192	100		82.5 (69.7–95.2)	188
	Germacrone (SO)	*Spodoptera littoralis* - L6	choice		50	90.5 ± 9.8		1.9 (0.1–3.6)	98
	Guaiacol	*Spodoptera littoralis* - L4	no choice		250	100			185
	Isoeugenol	*Spodoptera littoralis* - L4	no choice		250	100			185
	Pinocembrin	*Spodoptera frugiperda* - L3	choice		50	91			189
	Piperitenone	*Spodoptera littoralis* - L3	no choice		500 and 1000	84.0 and 100.0			190
	Piperitenone epoxide	*Spodoptera littoralis - L6*	choice		100			0.18 (0.01, 3.0)	99
	Pulegone	*Spodoptera littoralis - L6*	choice		50	100		0.2	61
	Pulegone	*Spodoptera littoralis - L6*	choice		100			0.25	99
	Plumbagin derivatives - **plumbagin+glycin (4a**); pl+methionine (4d)	***Spodoptera litura**; Achaea janata* – L3 both	no choice					**36.87**; 37.37	191
	Pregeijerene	*Spodoptera littoralis* - L3	choice		225	100		95.1 (83.3–107.0)	188
	Safrole	*Spodoptera littoralis* - L6	choice		50	80.5 ± 7.2		5.25 (1.3, 20.7)	62
	Salicyaldehyde	*Spodoptera littoralis* - L4	no choice		250	100			185
	Semisynthetic eugenol derivatives - eugenol, isoeugenol	*Spodoptera frugiperda* - L (unspecified)	no choice	1000		75 and 78.5			192
	Silphinene	*Leptinotarsa decemlineata* - A	choice					0.147	96
	Terpinolene	*Spodoptera littoralis* - L6	choice		50	73 ± 12.6		53.80 (26.8, 99.4)	62
	Thujone	*Spodoptera littoralis* - L6	choice		50	100		0.20	61

	Thymol	*Spodoptera littoralis* - L6	choice		50	78.5 ± 8.0		21.0 (14.5, 27.1)	61
	Thymol	*Spodoptera littoralis* - L4	no choice		250	100			185
	Trans-anethole	*Hyphantria cunea* - L4	no choice	1.41 ul mL^{-1}		87			193
	Trans-Ethyl cinnamate	*Spodoptera littoralis* - L3	no choice		500 a 1000	86.7 / 100.0			190
	2-etylphenol	*Spodoptera littoralis* - L4	no choice		250	100			185
	2, 6 - dimethoxyphenol	*Spodoptera littoralis* - L4	no choice		250	100			185
	2-methoxy-4-methylphenol	*Spodoptera littoralis* - L4	no choice		250	100			185
	4-ethylguaiacol	*Spodoptera littoralis* - L4	no choice		250	100			185
	4-ethylphenol	*Spodoptera littoralis* - L4	no choice		250	100			185
	11α-Epoxy-eremophil-9-en-8-one (ligudicin A)1	*Spodoptera littoralis* - L6	choice		50	89 ± 3		0.08 (0.04 – 0.18)	69
	(E)-β-Ocimene	*Spodoptera littoralis* - L6	choice		50	92.7		10.6 (7.1, 15.9)	100
